# RNA-Binding Peptide Influences Epitranscriptomic Regulation by Preferentially Binding to Unmodified RNAs Targeted by NSUN2

**DOI:** 10.3390/biom16091287

**Published:** 2026-09-05

**Authors:** Chathurani Ekanayake, Aftab Mollah, Maelee Thompson, Elizabeth Hout, Chamali Thalagaha Mudiyanselage, Sanjaya Abeysirigunawardena

**Affiliations:** Department of Chemistry and Biochemistry, Kent State University, Kent, OH 44242, USA; eekanay1@kent.edu (C.E.); amollah@kent.edu (A.M.); mthom203@kent.edu (M.T.); ehout2@kent.edu (E.H.); cthalaga@kent.edu (C.T.M.)

**Keywords:** 5-methylcytosine (m^5^C), epitranscriptomics, NSUN2, RNA-binding peptides, phage display, RNA modifications

## Abstract

5-Methylcytosine (m^5^C) is a widespread mRNA modification that regulates gene expression and is frequently dysregulated in cancer. Here, we used phage display to identify peptides that bind an NSUN2 consensus RNA sequence in either its unmodified (UN2-RNA) or m^5^C-modified (MN2-RNA) form. A single peptide, un2p1 (TDYSTHRLSHSL), was selectively enriched against both targets. Biophysical analyses demonstrated that un2p1 binds this RNA sequence with high affinity and that m^5^C incorporation reduces binding by approximately ninefold, indicating that cytidine methylation acts as a biochemical switch for peptide–RNA recognition. Sequence alignments mapped un2p1 to a conserved structural loop and catalytic domain region within NSUN2, suggesting that the peptide functions as a molecular mimic of the endogenous RNA-recognition interface. In A549 lung adenocarcinoma cells, un2p1 treatment reduced global m^5^C levels and downregulated the oncogenic chaperonin CCT5, consistent with disruption of NSUN2-dependent Wnt/β-catenin signaling. These effects were attenuated in non-malignant HEK293 cells, which exhibited a compensatory increase in m^5^C levels and preserved viability. Together, these findings identify un2p1 as a sequence-specific, methylation-sensitive RNA-binding peptide that modulates the m^5^C epitranscriptome and selectively impairs lung cancer cell viability, highlighting a substrate-centric strategy for targeting NSUN2-mediated oncogenic pathways.

## 1. Introduction

Nucleotide modifications, such as 5-methylcytosine (m^5^C), play a pivotal role in post-transcriptional regulation, including alternative splicing, translation control, and mRNA degradation [1,2]. These m^5^C modifications are dynamic: cytosine methylations are added by methyltransferases such as NSUN (Nop2/Sun), DNMT (DNA methyltransferase), and TRDMT (tRNA aspartic acid methyltransferase) [3] and removed or further modified by demethylases such as TET [4] and ALKBH1 [5]. While these methyltransferases (writers) and demethylases (erasers) maintain a dynamic equilibrium of m^5^C levels, proteins that recognize m^5^C modifications, collectively known as readers, determine the fate of m^5^C-modified RNAs [3]. In other words, m^5^C modifications can function as molecular switches that inhibit or promote RBP binding to specific m^5^C-modified RNA motifs, thereby directing the respective RNAs toward different fates, such as altered splicing, transport, degradation, or stabilization [6]. Disruption of interactions involving these readers has been associated with many non-communicable diseases and health conditions, among which non-small cell lung cancer (NSCLC) is particularly prominent. Such prominence is due to the association between increased m^5^C modifications and unfavorable clinical outcomes, mediated by the stabilization of several oncogenes, such as CCT5, which functions in the Wnt/β-catenin pathway [7,8]. However, there has not yet been adequate development of peptide-based or other small-molecule tools targeting m^5^C–RBP interactions to overcome cancer. In response to this gap in the development of drugs targeting epitranscriptomic machinery, this study explores the potential of RNA-binding small-molecule inhibitors of RNA methyltransferases as candidate therapeutics for cancer. Thus, we hypothesize that rationally designed, RNA-binding small-molecule inhibitors of m^5^C RNA methyltransferases can selectively disrupt oncogenic m^5^C-dependent RBP–RNA interactions and thereby attenuate m^5^C-driven malignant phenotypes in various cancers.

## 2. Materials and Methods

### 2.1. Oligonucleotide Preparation and Quantification

Chemically synthesized RNA and DNA oligonucleotides used in this study were purchased from Horizon Discovery (St. Louis, MO, USA) and Integrated DNA Technologies (Coralville, IA, USA), respectively. High-performance liquid chromatography (HPLC)-purified RNA oligonucleotides were obtained in a 2′-ACE-protected form. Deprotection was executed via incubation in a specialized 2′-deprotection buffer consisting of 100 mM acetic acid (pH 3.8, adjusted with TEMED) at 60 °C for 30 min, with the incubation period extended to 2 h for 5′-biotinylated RNA species. Deprotected RNA transcripts were subsequently dried by centrifugal vacuum concentration and stored at −20 °C. For experimental use, RNA pellets were reconstituted in TE buffer (10 mM Tris-HCl, pH 7.5, 1 mM EDTA) to a stock concentration of 100 µM. Absolute oligonucleotide concentrations were determined spectrophotometrically using a NanoDrop One (Thermo Scientific, Waltham, MA, USA) from absorbance at 260 nm (A_260_) and sequence-specific molar extinction coefficients. Synthetic DNA oligonucleotides purchased from IDT were resuspended in RNase-free water to a final concentration of 100 µM and stored at −20 °C.

### 2.2. Phage Display Selection

Affinity selection (biopanning; Figure S1) was carried out over four iterative selection cycles using an M13 phage library displaying randomized pIII-fused dodecamer peptides as previously described in detail [9,10,11]. Selection stringency was progressively elevated across subsequent rounds by systematically increasing both the total number of washing steps and the concentration of non-ionic detergent within the wash buffer to favor high-affinity binders. Negative counter-selection against streptavidin-coated magnetic beads and solid-phase polystyrene surfaces was integrated into the primary cycle to eliminate matrix-specific binders. Subsequent panning cycles incorporated random competitor oligonucleotides and target-adjacent RNA sequences to subtract distal-site and non-specific polyanionic interactors (Table S1). Eluted phages harvested from the fourth cycle were titered on indicator bacterial lawns (Figure S2), and approximately 60 discrete plaques were isolated for downstream genomic DNA extraction. The variable locus encoding the randomized peptide region was amplified by PCR using primers suggested by the manufacturer (Table S2), and the resulting amplicons were purified using an E.Z.N.A. Cycle Pure Kit (Omega Bio-Tek, Norcross, GA, USA) and structurally verified via 1.5% (*w*/*v*) agarose gel electrophoresis (Figure S3). Purified PCR products underwent high-throughput Sanger sequencing (GenScript, Piscataway, NJ, USA) to decipher the primary amino acid sequences of the enriched pIII-fused dodecamer peptides.

### 2.3. Bioinformatic Alignment and Cross-Species Conservation Analysis

To map the structural and evolutionary origins of the biopanning-selected un2p1 peptide sequence (TDYSTHRLSHSL), localized pairwise alignment was conducted against the primary sequence of the human epitranscriptomic RNA methyltransferase NSUN2 (*Homo sapiens*, UniProt ID: Q08J23). Pairwise alignments were executed utilizing the Basic Local Alignment Search Tool (BLAST) via the National Center for Biotechnology Information (NCBI) portal (Bethesda, MD, USA; https://blast.ncbi.nlm.nih.gov accessed on 2 September 2026), Jalview software (version 2.11.3, University of Dundee, Dundee, UK), utilizing its integrated CLUSTALW alignment engine. to optimize alignment scoring matrices. Sequence identity, quality tracks, and positional occupancy were evaluated to identify regions of localized sequence homology. To find out the evolutionary constraints acting upon this specific locus, multiple sequence alignments (MSA) were performed across divergent eukaryotic reference taxa. The comparative pool included mammalian (*Homo sapiens* and *Rattus norvegicus*), amphibian (*Xenopus laevis*), and invertebrate (*Drosophila melanogaster*) lineages. Evolutionary conservation profiles and scoring bars were calculated based on residue-by-residue physicochemical substitution metrics within the Clustal alignment suite to map the positional retention of the matching peptide loop across metazoan lineages.

### 2.4. Isothermal Titration Calorimetry (ITC)

Calorimetric titrations were performed on a MicroCal PEAQ-ITC (Malvern Panalytical, Westborough, MA, USA) using peptides synthesized by GenScript. For peptide-RNA binding, methylated or unmethylated target RNAs (20 µM) in Buffer F (50 mM Tris-HCl, pH 7.5, 150 mM NaCl) were titrated into un2p1 peptide (2 µM) in the same buffer. Equilibrium dissociation constants (K_D_) and thermodynamic parameters were calculated using a single-set-of-identical-sites binding model. All measurements were performed in triplicate, with reported errors representing the standard deviation (SD).

### 2.5. Mammalian Cell Culture

Human lung adenocarcinoma (A549), breast adenocarcinoma (MDA-MB-231), cervical carcinoma (HeLa), and human embryonic kidney (HEK293T) cells were purchased from the American Type Culture Collection (ATCC). Cell lines were maintained in high-glucose Dulbecco’s Modified Eagle Medium (DMEM; 4.5 g/L glucose, Corning) supplemented with 10% (*v*/*v*) heat-inactivated fetal bovine serum (FBS, Gibco) and 1% (*v*/*v*) penicillin–streptomycin antibiotic cocktail (Corning, Glendale, AZ, USA). Cultures were maintained in a humidified incubator chamber regulated at 37 °C under an atmosphere of 5% CO_2_. Upon reaching 70–80% active monolayer confluency, cell populations were passaged via enzymatic dissociation using 0.25% Trypsin–EDTA and re-seeded at a standard 1:5 splitting ratio for downstream functional, viability, and epitranscriptomic assays.

### 2.6. Peptide Treatment and Total RNA Extraction

A549 lung adenocarcinoma and HEK293T embryonic kidney cells were seeded in 6-well culture plates at an initial density calculated to reach approximately 50–80% active monolayer confluency on the day of treatment. Cells were subsequently treated with the synthetic un2p1 peptide (10 µM) using the TransIT-X2 transfection delivery system (Mirus Bio, Madison, WI, USA) to facilitate intracellular internalization. Total RNA was isolated from both treated and untreated control cell populations 48 h post-treatment using the E.Z.N.A.^®^ HP Total RNA Isolation Kit (Omega Bio-Tek, Inc., Norcross, GA, USA) in strict accordance with the manufacturer’s provided high-performance isolation protocol. Eluted total RNA matrices were immediately evaluated spectrophotometrically using a NanoDrop One UV-Vis spectrophotometer (Thermo Scientific). Absolute nucleic acid concentrations and purity profiles were determined based on sequence-specific molar extinction coefficients and standard A260/A280 absorbance ratio thresholds prior to downstream global m^5^C quantification and RT-qPCR gene expression analyses.

### 2.7. Global m^5^C RNA Methylation Quantification

Global N5-methylcytosine (m^5^C) levels within the isolated total RNA matrices from treated and untreated A549 and HEK293T cells were quantified utilizing the EpiQuik m^5^C RNA Methylation Elution/Quantification Kit (Colorimetric; Epigentek, Farmingdale, NY, USA), following the manufacturer’s established high-throughput protocol. Briefly, equal mass inputs of purified total RNA sample fractions were bound to the assay strip wells, followed by sequential incubations with the targeted m^5^C capture antibody and a biotinylated detection antibody. The relative presence of methylated cytosine residues was resolved calorimetrically by measuring the absorbance at 450 nm on a microplate reader. m^5^C levels were calculated based on the provided standards and normalized to total RNA input.

### 2.8. In Vitro Cytotoxicity Assays (MTT Assay)

Cells were seeded in 96-well plates (5 × 10^3^ cells/well) and incubated at 37 °C under 5% CO_2_ for 24 h before treatment with the un2p1 peptide (10 μM) for 48 h. Cell proliferation was quantified using the CellTiter 96^®^ Non-Radioactive Cell Proliferation Assay (Promega, Madison, WI, USA) according to the manufacturer’s protocol. Briefly, 20 μL of the provided MTT Dye Solution was added to each well and incubated at 37 °C for 4 h to allow formazan crystal formation. Absorbance was recorded at 570 nm using a microplate reader, and relative cell viability was calculated against untreated vehicle controls. To study the dose-dependent effects of the un2p1 peptide, cells were treated with the peptide (0–200 μM) upon reaching ~80% confluency using the TransIT^®^-X2 Dynamic Delivery System (Mirus Bio, Madison, WI, USA) alongside vehicle controls for 48 h. Then, 15μL of dye solution was added to each well and incubated at 37 °C for 4 h, and absorbance was recorded at 570 nm using a Agilent BioTek Synergy Neo2 microplate reader (Agilent Technologies, Santa Clara, CA, USA).

### 2.9. Statistical Analysis

All quantitative assays, including fluorometric binding titrations, global m^5^C determinations, and MTT viability values, were performed using at least three independent biological replicates. Data are presented as mean ± standard error of the mean (SEM). Statistical significance between experimental cohorts was calculated via unpaired Student’s *t*-tests or one-way analysis of variance (ANOVA) followed by Tukey’s post hoc tests using GraphPad Prism software (version 11.1.0, GraphPad Software, Boston, MA, USA). *p* < 0.05 was considered statistically significant.

### 2.10. Fluorometric RNA–Peptide Binding Assays

A constant concentration of N-terminally Cy3-labeled un2p1 peptide (50 nM) prepared in sterile, deionized aqueous solution was utilized. Titrations were executed by sequentially introducing increments of target RNA (either unmodified UN2 or methylated MN2) directly into the peptide solution. Following thorough mixing, steady-state fluorescence emission spectra were recorded upon excitation at the optimal absorption wavelength of the fluorophore. Raw intensity data were corrected for sample dilution, and the fractional change in fluorescence intensity (ΔF/F_0_) was plotted against total RNA concentration. Cooperative binding isotherms were analyzed and fitted via non-linear regression using the Hill equation to extract the apparent K_D_ and Hill coefficients (*n*).

## 3. Results

### 3.1. The Same Peptide Sequence Was Enriched in Phage Display for Both m^5^C-Modified and Unmodified RNAs but with Different Percentage Enrichment

**Figure 1:**
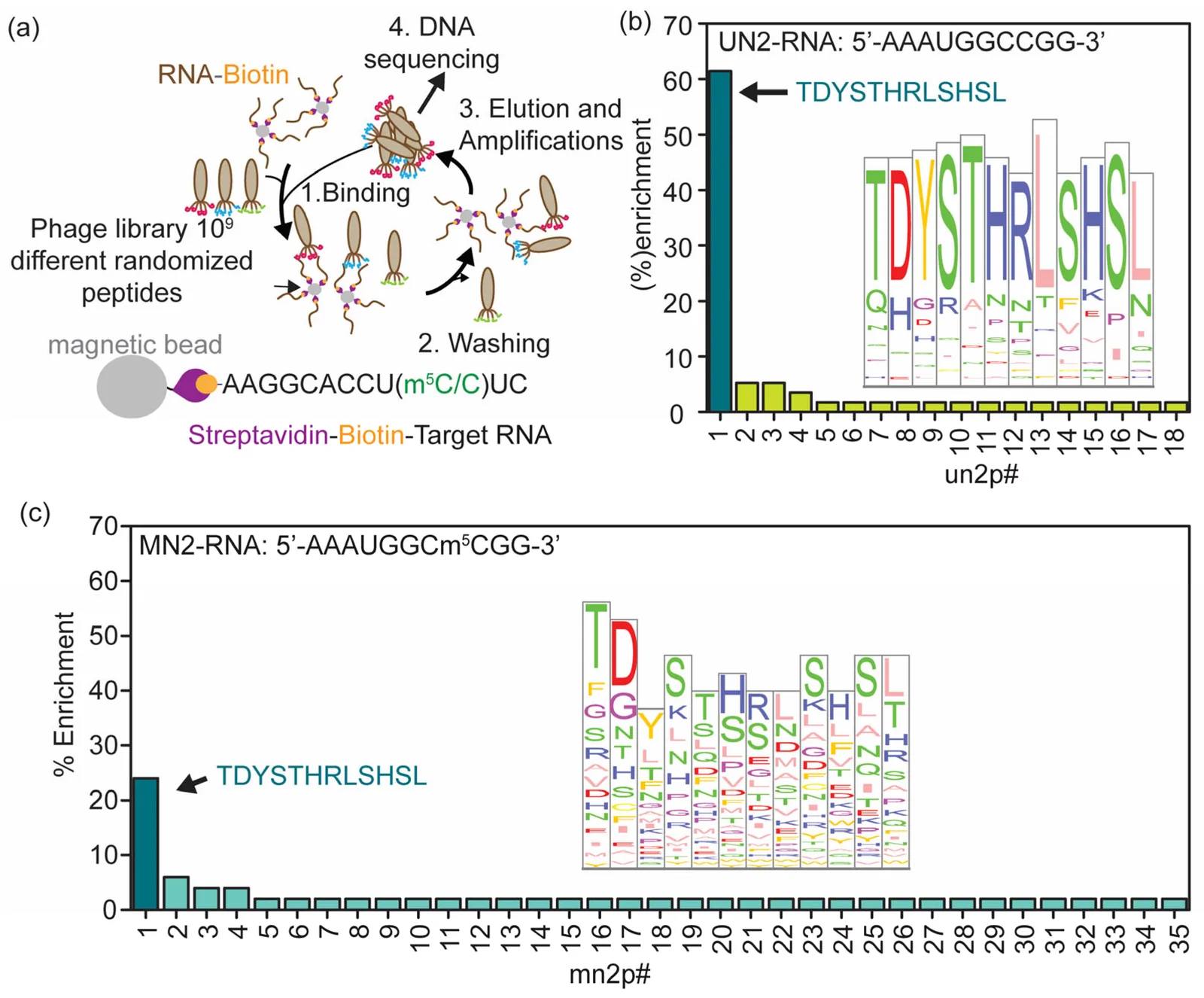
(**a**) Schematic of a phage display biopanning cycle. An M13 phage library consisting of 10^9^ unique phages with different dodecamer peptide sequences displayed on its pIII coat protein was screened against a biotinylated RNA target immobilized on streptavidin-coated magnetic beads. The percentage enrichment of peptides enriched in phage display experiments performed using (**b**) UN2-RNA and (**c**) MN2-RNA model RNAs is shown. Sequence logos, drawn in Clustal colors, for all enriched peptides against each target RNA are shown in the inset of the respective histogram.

To discover peptide-based small-molecule inhibitors that disrupt the modification machinery, phage display, a directed evolution technique, was used. Phage display selectively enriches phages with randomized peptide sequences fused to the pIII coat protein that specifically bind a target molecule [9,10,11,12,13]. Each phage display consisted of four biopanning cycles, during which the phage library (10^9^-fold sequence diversity) was screened against a target molecule (Figure 1a). In each biopanning cycle, a diverse pool of phages was incubated, with the biotinylated target molecules immobilized on streptavidin-coated magnetic beads. Stringency was progressively increased in each round by increasing the number of washing steps and the detergent concentration in the wash buffer to ensure retention of high-affinity binders [14]. Furthermore, counter-selection with random competitors was integrated into each cycle to eliminate non-specific binders and increase the selectivity of the enriched phage pool. Following the final biopanning cycle, phage DNA from individual colonies was isolated and sequenced by Sanger sequencing [15]. In this study, we performed two separate phage display experiments using two model RNAs, UN2 (AAAUGGCCGG), an RNA containing the consensus NSUN2-binding sequence, and its m^5^C-modified RNA counterpart MN2 (AAAUGGCm^5^CGG) as target molecules, to identify protein sequence motifs that enable m^5^C recognition. Using a single standard RNA target can be justified by the emerging evidence that NSUN2 acts on single standard regions of many RNAs [16]. A significant enrichment of a unique peptide sequence was observed in phage displays against both m^5^C-modified (MN2) and unmodified RNA (UN2) targets (Figure 1b,c, Tables S3 and S4). The un2p1 peptide (TDYSTHRLSHSL) was the most enriched peptide in both phage display experiments and showed an approximately 3-fold higher percentage enrichment for unmodified UN2 RNA (61%) than for m^5^C-modified MN2 RNA (24%).

### 3.2. Binding Affinities of the un2p1 Peptide to Model RNAs Are Comparable to the Percentage Enrichment of the Peptide to the Respective RNAs

**Figure 2:**
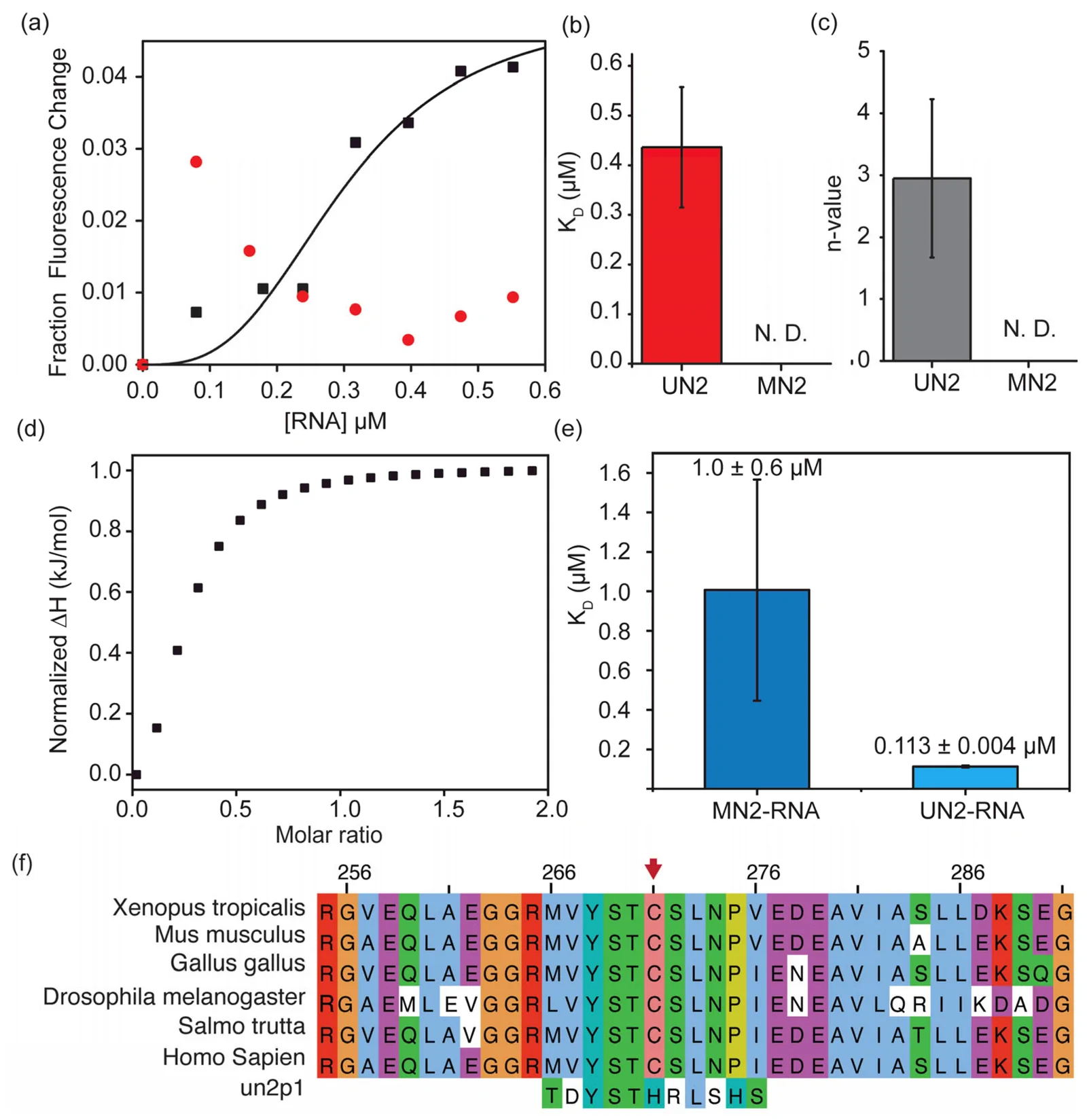
Biophysical characterization and evolutionary conservation of un2p1 peptide binding to target RNAs: (**a**) Steady-state fluorometric titration curves of N-terminally Cy3-labeled un2p1 (50 nM) with unmodified UN2 RNA (black squares) or m^5^C-modified MN2 RNA (red circles), fitted with the Hill equation. (**b**) K_D_s and (**c**) Hill coefficients (n) from fluorometric titrations. (**d**) Representative normalized ITC binding isotherm for un2p1–RNA interactions. (**e**) ITC-derived K_D_s showing reduced affinity for MN2 RNA (1.0 ± 0.5 μM) compared with UN2 RNA (0.113 ± 0.004 μM). Error bars represent the standard deviation of independent biological replicates. (**f**) Multiple sequence alignments of divergent metazoan NSUN2 orthologs with the un2p1 sequence; residue positions are numbered relative to consensus sequences. The red arrow indicates the catalytic cysteine.

While phage display is primarily a qualitative enrichment tool, fluorometric assays and isothermal titration calorimetry (ITC) were used to quantitatively assess binding affinity and characterize the thermodynamic profile of RNA–peptide interactions. For the fluorometric assay, the un2p1 peptide was conjugated to a Cyanine 3 (Cy3) dye at the N-terminus (Figure 2a). The change in fluorescence signal was recorded with the addition of unlabeled RNA. The signal decreased with the addition of UN2 RNA, whereas the fluorescence signal initially increased for MN2 RNA and then gradually decreased, indicating distinct un2p1 binding mechanisms for m^5^C-modified and unmodified RNAs. The fractional change in fluorescence, corrected for volume variations during titration, was plotted against UN2 RNA concentration, and the binding curves were fitted to the Hill equation (Figure 2a). The mean K_D_ obtained from three biological replicates for the peptide–RNA complex between un2p1 and UN2 RNA is 0.4 ± 0.1 μM (Figure 2b), and the Hill coefficient was determined to be 2 ± 1 (Figure 2c). Due to variability in the fluorescence change parameters, the binding characteristics for MN2 RNA could not be reliably ascertained, although binding was clearly detectable for both RNAs.

These ITC experiments were performed using a MicroCal PEAQ-ITC calorimeter (Malvern Panalytical, Westborough, MA, USA) (Figure 2d, Figures S4 and S5). In these experiments, the respective RNA in TK buffer (50 mM Tris-HCl, pH 7.5, and 150 mM KCl) was titrated onto 2 µM peptide in the same buffer. The heat released upon adding the respective RNA to un2p1 indicates interactions between RNA and peptide. These binding curves were fitted to a binding model that assumes a single set of identical binding sites in the peptide. The MN2–RNA–un2p1 complex had a K_D_ of 1.0 ± 0.6 μM (Figure 2e). Calorimetric titrations with unmethylated UN2 RNA and un2p1 produced a K_D_ of 0.113 ± 0.004 μM, indicating a higher binding affinity for the peptide in the absence of m^5^C modification on RNA (Figure 2e, Table S5).

### 3.3. The Sequence of the un2p1 Peptide Aligns with Two Regions of NSUN2 Proteins

To elucidate the sequence origin and biological relevance of the un2p1 sequence, local alignments were performed against the human NSUN2 methyltransferase sequence and subsequently aligned across divergent eukaryotic taxa (Figure 2f). Pairwise local alignment of the 12-mer peptide un2p1 against the primary sequence of human RNA methyltransferase NSUN2 (*Homo sapiens*, UniProt ID: Q08J23) identified a highly conserved sequence motif. The un2p1 (TDYSTHRLSHSL) peptide maps to an endogenous structural loop within human NSUN2 spanning residues 78–89 (Figure 2f). In addition, the un2p1 sequence aligns with a region (266–276) within the methyltransferase domain (MTD), the catalytic domain of NSUN2 (Figure S6). Sequence alignment scores confirmed high evolutionary conservation and optimal sequence consensus at these positions. Multiple sequence alignments (MSAs) across phylogenetically distant model organisms, including Xenopus laevis, Drosophila melanogaster, and *Rattus norvegicus*, showed that 12-residue segments similar to the un2p1 motif exhibit high evolutionary conservation across all taxa analyzed (Figure 2f and Figure S6).

### 3.4. The un2p1 Peptide Inhibits NSUN2, Leading to Decreased m^5^C Levels and CCT5 mRNA Levels in Lung Adenocarcinoma Cells, Lowering Their Viability

**Figure 3:**
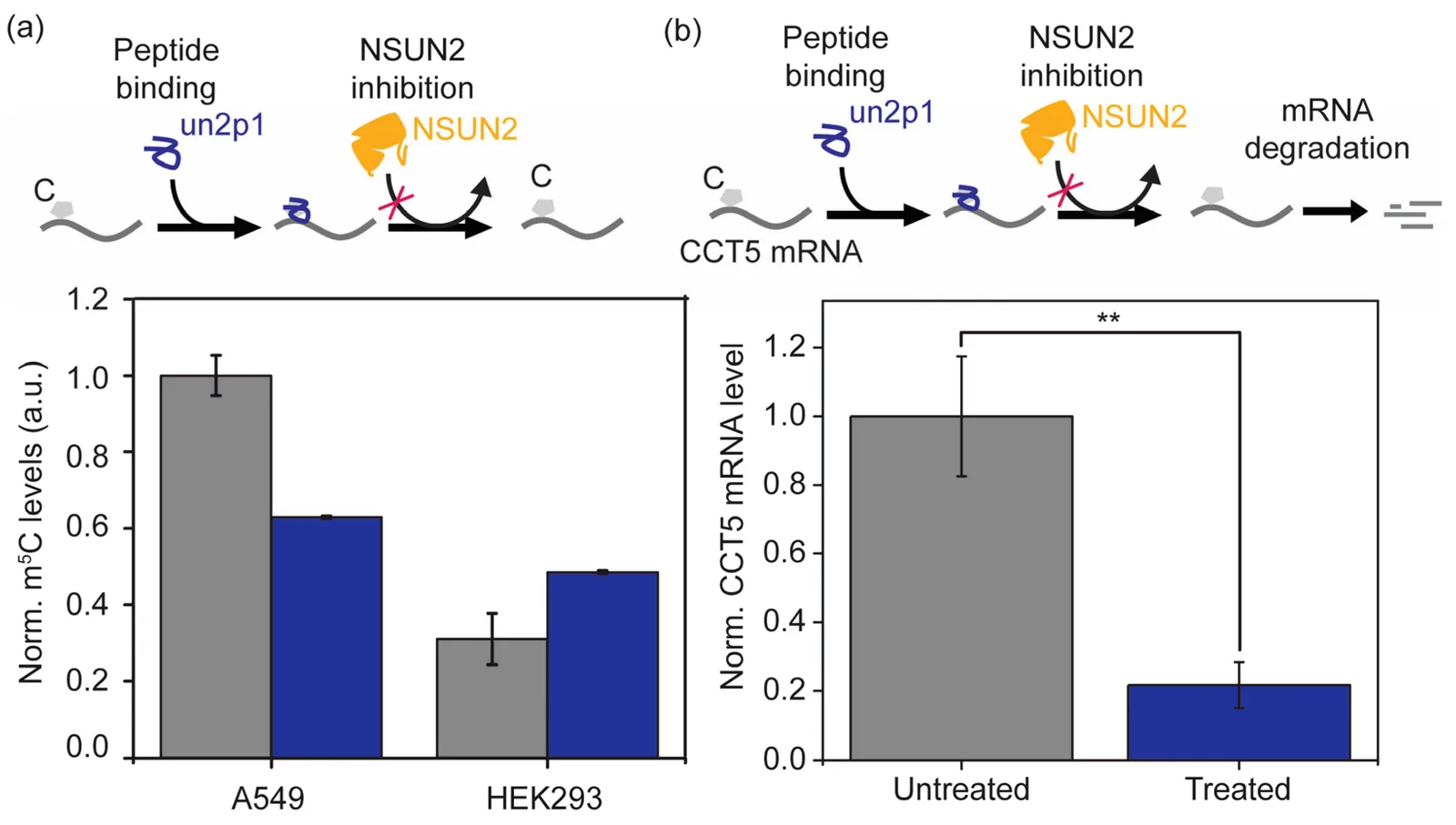
The un2p1 peptide modulates global m^5^C levels and CCT5 expression in human cells: (**a**) Normalized global m^5^C levels in A549 lung adenocarcinoma and HEK293T cells following treatment with un2p1 peptide (10 µM) (blue bars) compared with untreated controls (grey bars). un2p1 treatment leads to a significant decrease in m^5^C levels in A549 cells, whereas HEK293T cells exhibit a compensatory increase, indicating cell line–specific regulation of RNA methyltransferase activity. Errors represent the standard deviation from three biological replicates. (**b**) Quantitative RT–PCR analysis of CCT5 mRNA levels in A549 cells treated with un2p1. Peptide treatment results in an approximately 80% reduction in CCT5 transcript levels relative to untreated controls. The double asterisk (**) denotes statistical significance at *p* < 0.01 relative to the untreated control group, calculated via an unpaired Student’s t-test.

**Figure 4:**
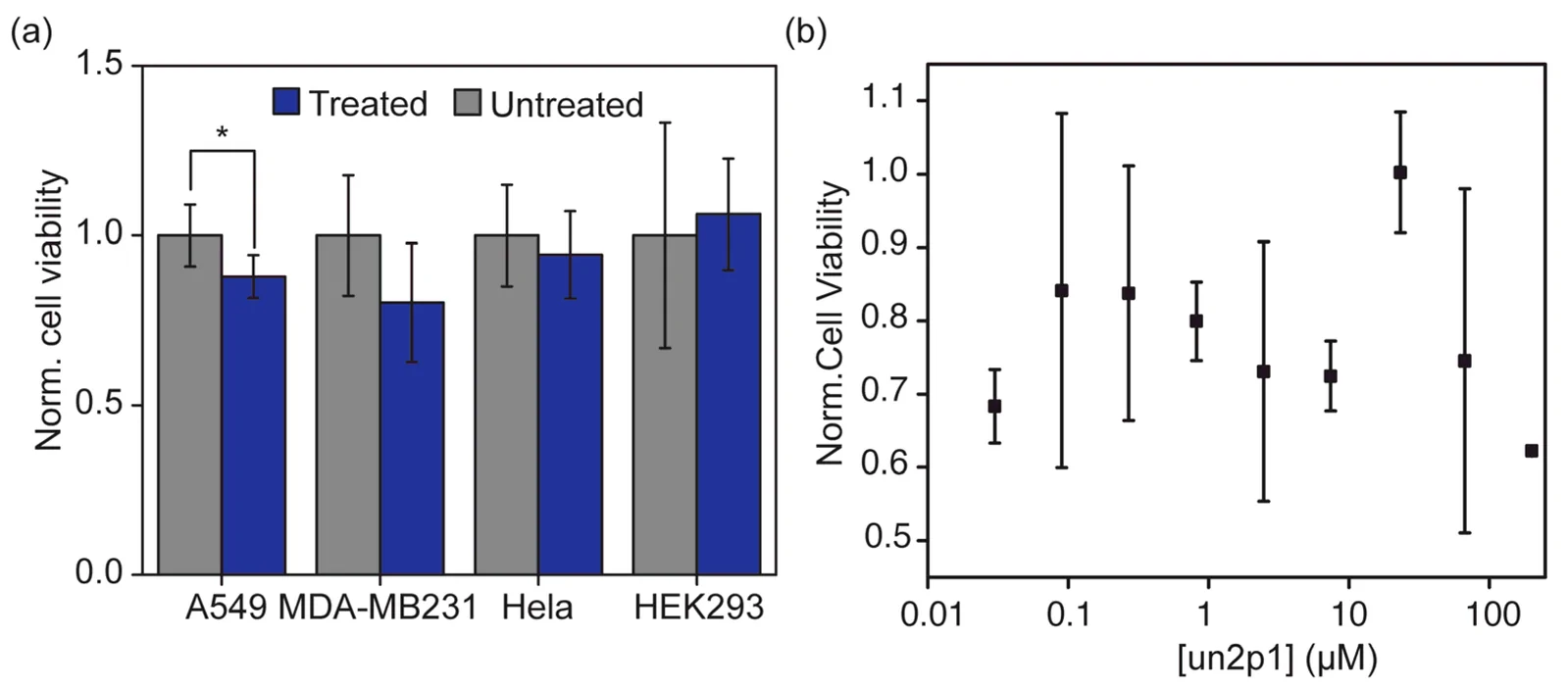
The un2p1 peptide influences the viability of A549 lung adenocarcinoma cells. (**a**) Normalized cell viability of A549, MDA-MB-231, HeLa, and HEK293 cells after un2p1 peptide (10 µM) treatment is shown, with treated cells (blue) compared to untreated controls (gray). Cell viability was assessed by MTT assay 48 h after peptide transfection and is expressed relative to untreated controls. Error is calculated as standard deviation from three independent biological replicates. A significant reduction in viability is observed in A549 cells; the single asterisk (*) denotes statistical significance at *p* < 0.05 relative to the untreated control group. (**b**) Dose–response of the un2p1 peptide for A459 cells is shown.

m^5^C levels in total RNA extracted from A549 lung adenocarcinoma cells and HEK293, a control cell line, following un2p1 peptide treatment were quantified to investigate the ability of the peptide to inhibit NSUN2 enzymatic activity (Figure 3a). In A549 cells, peptide treatment resulted in a 38% decrease in m^5^C levels. In contrast, m^5^C levels in total RNA extracts from HEK293 cells increased by 57%. RT-qPCR analysis of CCT5 following treatment with un2p1 was performed to evaluate the functional consequences of inhibited NSUN2-mediated activity (Figure 3b). un2p1-treated A549 cells showed an approximately 80% decrease in CCT5 mRNA expression (Figure 3b). The survival of four cell types, A549, MDA-MB, HeLa, and HEK293T, after treatment with the un2p1 peptide was investigated to assess the efficacy of un2p1 as a potential anticancer therapeutic agent (Figure 4). The un2p1 peptide was added after the cells adhered to a 96-well plate and reached greater than 80% confluency. Cell viability was measured using a standard MTT-based assay. Cell viability decreased significantly in A549 lung adenocarcinoma cells (to 12.06% of control, *p* = 0.026087), whereas the decrease in viability was not significantly different in MDA-MB and HeLa cells. The normalized cell viability for non-cancerous HEK293 cells remained close to or above 1.0 (Figure 4a). Dose–response experiments were performed in A549 cells to evaluate the inhibitory potency (IC50) of un2p1 (Figure 4b). At lower peptide concentrations, cell viability decreased compared with the untreated cells. However, at mid-range concentrations of the peptide (50 μM > [un2p1] > 10 μM), cell viability increased, followed by a gradual drop. These consistently observed opposing trends precluded reliable least-squares fitting of a standard sigmoidal dose–response curve, and an accurate IC50 value could not be determined. The overall A549 cell viability was reduced to approximately 85% at a concentration of 67 μM of un2p1. Such biphasic instances are well-documented across small molecules and biopeptides, often preventing standard sigmoidal IC50 curve fitting [17,18,19].

## 4. Discussion

The phage display selection identified un2p1 as a unique peptide that is strongly enriched against both unmodified UN2 and m^5^C-modified MN2 target RNAs. The approximately threefold higher enrichment observed for UN2 compared with MN2 suggests that cytidine methylation within the NSUN2 consensus sequence functions as a biochemical “switch” that perturbs peptide–RNA interactions. These observations indicate that, although un2p1 is sequence-specific, it is highly sensitive to the methylation status of the cytidine residue and that m^5^C within the NSUN2-binding sequence can serve as a negative regulator of protein recruitment. Two major potential factors, steric hindrance and conformational modulation, may account for the low preference for m^5^C-modified RNAs. Loss or weakening of short-range interactions, as an indirect result of m^5^C modification, can lower the peptide’s affinity for RNA. In addition, m^5^C modification is known to alter RNA base-stacking interactions [20,21], likely reorganizing the spatial orientation of the phosphate backbone or neighboring bases, thereby rendering the target RNA’s binding surface less complementary and reducing accessibility to the un2p1 peptide.

Fluorometric and ITC analyses further support a model in which un2p1 binds with higher affinity to unmethylated RNA than to its m^5^C-modified counterpart. The submicromolar K_D_ for UN2 RNA and a higher K_D_ for MN2 RNA indicate a pronounced reduction in binding affinity upon cytidine methylation, consistent with the “switch-like” effect inferred from the phage display enrichment data. The cooperative binding behavior observed in the fluorometric titrations (Hill coefficient ~2) suggests that un2p1 may engage multiple binding sites or induce higher-order RNA–peptide assemblies, although the precise mechanistic basis of this cooperativity remains to be elucidated. The inability to reliably determine binding parameters for MN2 in the fluorometric assay, despite clear evidence of interaction, further underscores that m^5^C alters the binding energy landscape rather than abolishing binding altogether.

Bioinformatic analyses provide insight into the structural and evolutionary context of the un2p1 motif. Local alignment of the un2p1 sequence to human NSUN2 reveals that this peptide maps to two distinct regions: a structural loop (residues 78–89) and a segment within the methyltransferase domain (residues 266–276). The high sequence conservation of these regions across diverse eukaryotic lineages, from amphibians to mammals, suggests that they are under stringent evolutionary constraint. This conservation implies that the corresponding domains are thermodynamically or kinetically essential for maintaining the NSUN2 tertiary structure, substrate positioning, or catalytic turnover. Accordingly, the un2p1 peptide can be viewed as a molecular mimic of an endogenous regulatory interface or substrate-recognition topology inherent to the wild-type enzyme. By reproducing key features of the NSUN2–RNA recognition interface, synthetic un2p1 can competitively sequester the target RNA sequence motif and antagonize NSUN2 binding.

The ability of un2p1 to bind both unmodified and m^5^C-modified RNAs establishes a dual substrate-masking mechanism that has important implications for epitranscriptomic regulation. On one hand, un2p1 binding to the consensus NSUN2 sequence in unmodified RNA can prevent assembly of the NSUN2–RNA complex, thereby limiting the addition of new m^5^C modifications and reducing global methylation levels. On the other hand, residual or weak binding of un2p1 to m^5^C-modified RNA can block recognition of m^5^C methylations by m^5^C reader proteins and potentially impede demethylation by m^5^C erasers. Thus, un2p1 can simultaneously inhibit writer- and reader/eraser-dependent steps, thereby attenuating m^5^C-driven signaling in a context-dependent manner.

The divergent responses of A549 and HEK293 cells to un2p1 treatment highlight the importance of cellular context in shaping the net epitranscriptomic outcome. In A549 cells, the 38% reduction in global m^5^C levels suggests robust inhibition of endogenous methyltransferase activity, consistent with effective competition between un2p1 and NSUN2 for access to consensus RNA sites. Interestingly, previous reports have identified several m^5^C-dependent pathways implicated in cancer progression. For example, NSUN2 acts as an oncogene in lung adenocarcinoma by adding m^5^C modifications onto CCT5 mRNA, which recruits YBX1, a reader protein that stabilizes the CCT5 transcript and upregulates its expression. Consequently, inhibition of NSUN2 will decrease CCT5 levels, suppressing tumor cell proliferation, migration, and invasion [7]. The concomitant ~80% decrease in CCT5 mRNA further supports the idea that un2p1 disrupts NSUN2-dependent stabilization of CCT5 transcripts, leading to increased mRNA turnover. Because CCT5 functions as a chaperonin required for proper folding and maturation of β-catenin, decreased CCT5 expression is expected to promote proteasomal degradation of misfolded β-catenin and suppress Wnt/β-catenin signaling. These findings align with prior reports linking elevated m^5^C levels and NSUN2 overexpression to non-small cell lung cancer (NSCLC) pathogenesis [22,23,24]. In contrast, HEK293 cells exhibit a 57% increase in global m^5^C levels upon un2p1 treatment, accompanied by preserved or slightly increased viability. This pattern suggests that, rather than suppressing methyltransferase activity, un2p1 may stabilize methylated RNA or protect it from demethylation and degradation in this non-malignant cellular environment. A compensatory rise in m^5^C could constitute a stress-adaptive response that maintains transcript stability and cellular homeostasis. Together, these observations indicate that the net effect of un2p1 on the m^5^C-modified transcriptome is highly dependent on the relative expression levels of m^5^C writers, erasers, and readers, the abundance of target transcripts bearing the consensus sequence, and the basal methylation state of each cell type.

Treatment with un2p1 resulted in a statistically significant decrease in cell viability in A549 cells. In contrast, under the conditions tested, the observed changes in cell viability for MDA-MB-231, HeLa, and HEK293 cells did not reach statistical significance. In A549 cells, the biphasic dose–response to un2p1, with increased viability at lower concentrations and decreased viability at higher concentrations, suggests that the peptide may engage distinct mechanisms at different concentration ranges. For example, low-level binding could transiently modulate stress-signaling or survival pathways, while higher doses predominantly disrupt NSUN2-dependent oncogenic programs. The inability to determine a precise IC50, due to this biphasic behavior, underscores the complexity of un2p1’s mechanisms of action and suggests that additional optimization of delivery, stability, or sequence composition may be required to achieve more uniform cytotoxic responses. Limited cellular uptake of the peptide may further constrain maximal efficacy.

Compared with existing small-molecule NSUN2 inhibitors, un2p1 offers a conceptually distinct, substrate-centric strategy. Covalent probes such as MY-1B and targeted protein degraders such as GSK-F1 act primarily by reducing NSUN2 catalytic activity through engagement of the conserved active site [25,26]. Although such agents can effectively perturb the epitranscriptomic landscape and sensitize cancer cells to other therapies, their non-selective inhibition of NSUN2 also risks disrupting essential processes in non-malignant tissues, including translation by inhibiting tRNA methylation [27,28,29,30,31]. Furthermore, the high conservation of catalytic residues across the NSUN family raises concerns about cross-reactivity and off-target toxicity [29,30], potentially narrowing the therapeutic window and necessitating higher in vitro concentrations to achieve measurable effects. By contrast, un2p1 targets specific RNA sequences and structural motifs rather than the NSUN2 catalytic pocket itself. This substrate-centric approach may confer improved selectivity toward NSUN2-dependent oncogenic pathways that rely on methylation of particular transcripts, such as CCT5. The preferential impairment of A549 cell viability, coupled with the preservation of HEK293 cell viability, supports the feasibility of selectively disrupting NSUN2–RNA interactions in cancer cells without inducing systemic toxicity. At the same time, the context-dependent increase in m^5^C levels in HEK293 cells highlights the need for careful evaluation of potential compensatory responses in different tissues.

## 5. Conclusions

In this study, we have discovered a peptide sequence that binds to the NSUN2-binding RNA sequence. The presence of m^5^C modification within the sequence decreases the percentage enrichment and the affinity of the peptide (un2p1) to the RNA, highlighting the ability of RNA methylation to modulate RNA–protein interactions. Applying treatment of this peptide to A549 adenocarcinoma cells decreases the NSUN2 enzyme activity by substrate inhibition and thus decreases cellular m^5^C levels. Perhaps the viability of A549 cells decreases upon transfection of un2p1, partially or fully due to the lowering of oncogenic protein levels such as CCT5.

Overall, these findings identify un2p1 as a sequence-specific, methylation-sensitive RNA-binding peptide that can alter the m^5^C-modified transcriptome and selectively impair viability of A549 cells. By functioning as a molecular mimic of NSUN2’s NA-recognition interface and operating through a dual substrate-masking mechanism, un2p1 offers a promising proof of concept for targeting NSUN2-mediated oncogenic pathways via RNA-centered, rather than enzyme-centered, strategies. Future work will be required to dissect the structural basis of un2p1–RNA recognition, refine its sequence for enhanced potency and selectivity, and evaluate its efficacy and safety in more complex in vivo cancer models.

## Data Availability

The data supporting the findings of this study are provided within the article and the Supplementary Materials. All metadata will be available upon request from the corresponding author, in accordance with institutional and federal regulations.

## Supplementary Materials

The following supporting information can be downloaded at https://www.mdpi.com/article/10.3390/biom16091287/s1, Figure S1: Overview of the phage display biopanning workflow for RNA-binding peptide selection; Table S1: Biopanning selection stringency parameters across four iterative rounds; Figure S2: Representative agar plate showing phage plaque-forming units (PFUs) post-selection; Table S2: Oligonucleotide primer sequences for PCR amplification; Figure S3: Agarose gel electrophoresis of PCR-amplified phage insert DNA; Table S3: Sequence abundance and predicted physicochemical properties of peptides enriched against unmodified RNA targets; Table S4: Sequence abundance and predicted physicochemical properties of peptides enriched against unmodified RNA targets; Figure S4: Isothermal titration calorimetry (ITC) thermograms for un2p1 binding to MN2 RNA; Figure S5: Isothermal titration calorimetry (ITC) thermograms for un2p1 binding to UN2 RNA; Table S5: Thermodynamic binding constants determined by ITC for un2p1 interactions with target RNAs; Table S6: BLAST alignment for human proteins homologous to un2p1; Figure S6: Secondary alignment site for un2p1 peptide in NSUN2 of different eukaryotes is shown.

## Abbreviations

The following abbreviations are used in this manuscript:

NSUN2	NOP2/Sun RNA methyltransferase 2
CCT5	Chaperonin containing TCP1 subunit 5
