# Supplementary material for: RNA-Binding Peptide Influences Epitranscriptomic Regulation by Preferentially Binding to Unmodified RNAs Targeted by NSUN2
Source: Biomolecules. 2026 Sep 5;16(9):1287. doi: 10.3390/biom16091287 (PMC13604626; doi:10.3390/biom16091287)
Supplement: Supplementary file 1 [file biomolecules-16-01287-s001.zip › biomolecules-4498238-supplementary.pdf]

Supporting Information for

RNA-binding peptide influences epitranscriptomic regulation by preferably binding to unmodified RNAs targeted by NSUN2.

Chathurani Ekanayake [1], Aftab Mollah [1], Maelee Thompson [1], Elizabeth Hout [1], Chamali Thalagaha Mudiyanselage [1], and Sanjaya Abeysirigunawardena [1*]

1 Department of Chemistry and Biochemistry, Kent State University, Kent, Ohio, 44242, USA

* To whom correspondence should be addressed. Tel: +1 (330) 672-2667; Fax: +1 (330) 672 3816; Email: sabeysir@kent.edu

This PDF file includes:

Figure S1: Overview of the phage display biopanning workflow for RNA-binding peptide selection

Table S1: Biopanning selection stringency parameters across four iterative rounds

Figure S2: Representative agar plate showing phage plaque-forming units (PFUs) post-selection.

Table S2: Oligonucleotide primer sequences for PCR amplification

Figure S3: Agarose gel electrophoresis of PCR-amplified Phage inserts DNA.

Table S3: Sequence abundance and predicted physicochemical properties of peptides enriched against unmodified RNA targets.

Table S4: Sequence abundance and predicted physicochemical properties of peptides enriched against unmodified RNA targets

Figure S4: Isothermal Titration Calorimetry (ITC) thermograms for un2p1 binding to MN2 RNA.

Figure S5: Isothermal Titration Calorimetry (ITC) thermograms for un2p1 binding to UN2 RNA

Table S5: Thermodynamic binding constants determined by ITC for un2p1 interactions with target RNAs.

Table S6: BLAST alignment for human proteins homologous to un2p1

Figure S6: Secondary alignment site for un2p1 peptide in NSUN2 of different eukaryotes is shown

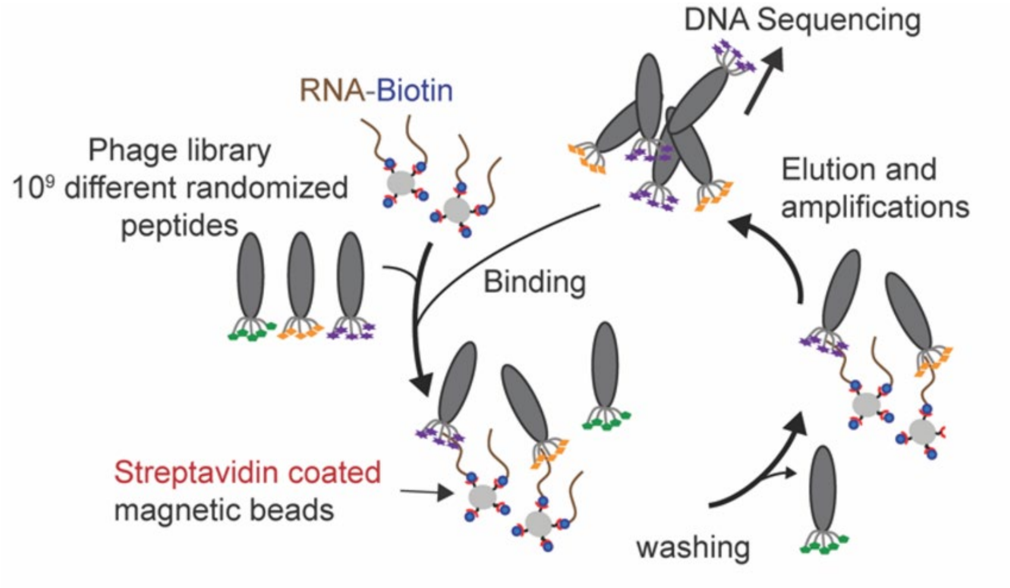

Figure S1: Overview of the phage display biopanning workflow for RNA-binding peptide selection.M13 phage library displaying 10^9 randomized peptides against biotinylated target RNA immobilized on streptavidin-coated magnetic beads. The selection cycle involves peptide binding, washing steps to remove non-specific binders, elution, and amplification of selected phages. High throughput sequencing follows to identify enriched peptide candidates.

Table S1: Biopanning selection stringency parameters across four iterative rounds. Summary of experimental conditions used during each biopanning cycle. Selection stringency was progressively increased by varying detergent concentration (0.1%–0.3% Tween-20), increasing the total wash count (4 to 8 washes) and introducing 50 nM competitor RNA in the final round to isolate high-affinity target binders.

**Conditions**	**Biopanning cycle**			
	**1st**	**2nd**	**3rd**	**4th**
Target RNA	50nM	50nM	50nM	50nM
Tween20	0.2%	0.1%	0.3%	0.3%
# of washes (1mL/wash)	4	2	6	8
Competitor RNA	-	-	-	50nM

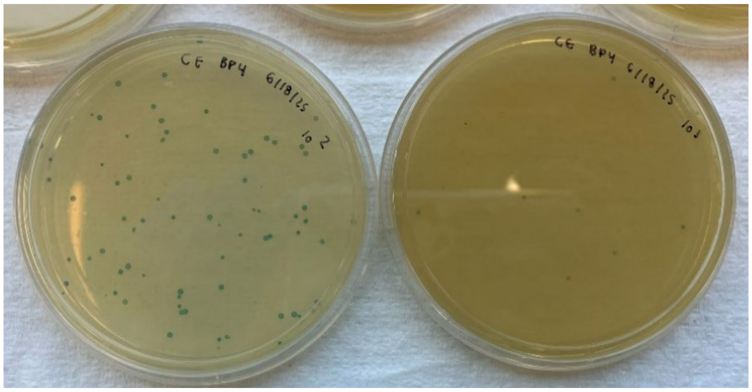

Figure S2: Representative agar plate showing phage plaque-forming units (PFUs) post-selection. Top-down view of LB/IPTG X-Gal plates displaying blue plaques resulting from serial dilutions of phage-infected *E. coli*. Plaque counts provide an empirical estimate of viral titer to verify successful phage enrichment prior to downstream amplification.

Table S2: Oligonucleotide primer sequences for PCR amplification. DNA sequences of the forward and reverse primers used to amplify the variable peptide-coding region from recovered phage libraries prior to sequencing.

**Forward primer**	5-GCA ATT CCT TTA GTG GTA CCT TTC-3
**Reverse primer**	5-CCC TCA TAG TTA GCG TAA CG-3

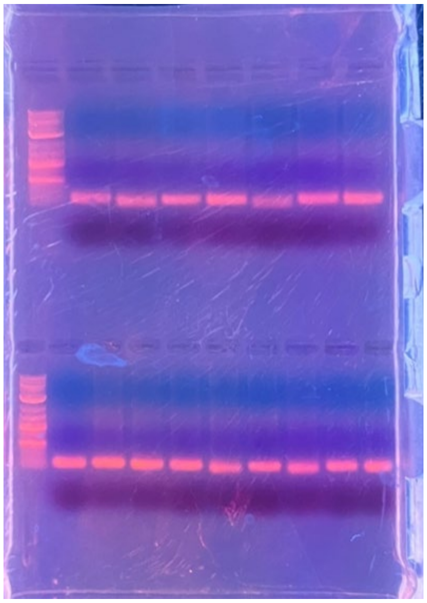

Figure S3: Agarose gel electrophoresis of PCR-amplified Phage inserts DNA.UV-transilluminated agarose gel displaying PCR amplification products corresponding to the target variable region of isolated phage clones prior to purification. Clear uniform bands confirm specific amplification across independent samples alongside molecular weight markers.

Table S3: Sequence abundance and predicted physicochemical properties of peptides enriched (n=50) for unmodified RNA targets. Sequence composition, occurrence frequency (%), and calculated biophysical parameters (isoelectric point [pI], grand average of hydropathicity [GRAVY], and molar extinction coefficient) for peptide clones isolated following selections against unmodified target RNAs.

Peptide	% Occurrence	PI	GRAVY	Extinction Coefficient
TDYSTHRLSHSL	61.40350877	6.61	−0.992	1490
QHDSANTLVEPN	5.263157895	4.35	−1.167	0
QHGRTPNTFKIN	5.263157895	11	−1.517	0
TDYSANPLVVPQ	3.50877193	4.05	−0.208	1490
TDYSTHRLLHSL	1.754385965	6.61	−0.608	1490
THYSTHSLLHSL	1.754385965	6.73	−0.275	1490
TESITDAPGGSS	1.754385965	4.05	−0.575	0
QHGRIANTFKIN	1.754385965	11	−0.800	0
NDYCNYNLFHSN	1.754385965	5.08	−1.250	2980
HHLQLSFRDSDG	1.754385965	5.98	−1.083	0
NDYSTHRLSHSL	1.754385965	6.92	−1.225	1490
QSHRDSTFGTSL	1.754385965	6.74	−1.025	0
SDYSTHRLSHSL	1.754385965	6.66	−1.000	1490
THYRTHRLSHSI	1.754385965	10.83	−1.217	1490
PDYSTHRLSHSL	1.754385965	7.33	−1.067	1490
THYSTHRLSHSI	1.754385965	8.49	−0.908	1490
QHHRTHSLCNSI	1.754385965	8.28	−1.050	0
TDQSTHXLSHSN	1.754385965	5.9	-1.7	0

Table S4: Sequence abundance and predicted physicochemical properties of peptides enriched (n=57) against unmodified RNA targets. Sequence composition, occurrence frequency (%), and calculated biophysical parameters (isoelectric point [pI], grand average of hydropathicity [GRAVY], and molar extinction coefficient) for peptide clones isolated following selections against modified target RNAs.

Sequence	Occurrence (%)	pI	GRAVY	Extinction Coefficient
TDYSTHRLSHSL	24	6.61125	-0.99167	1490
TGTLLVSNKLLT	6	8.41011	0.725	0
RTMNQFEDFFQP	4	4.370259	-1.16667	0
FCLHGSSNCGTH	4	6.898344	-0.11667	0
FGEVLEKRIYRT	2	8.588946	-0.58333	1490
TGTITVSNKLXT	2	8.41011	-0.2	0
STGNKLVKDLAT	2	8.310378	-0.31667	0
FAAKSPVDRPIQ	2	8.747731	-0.35833	0
GTATHTRLTTPA	2	9.756532	-0.48333	0
TDRNAIGPSEAL	2	4.368156	-0.55	0
YINHEPFGVYNR	2	6.747777	-0.94167	2980
IDPKFHDYQVAI	2	5.210851	-0.225	1490
SHFGSSHEIHGS	2	6.195302	-0.81667	0
SSLRMLSMDFQL	2	5.552965	0.341667	0
THKPDTSWLTLR	2	8.438799	-1.075	5500
EFLSNNSTSLTQ	2	4.050028	-0.61667	0
ADLGNSWMGRKH	2	8.798597	-1.13333	5500
VITHHDSVATEH	2	5.717741	-0.34167	0
DEFMMYKSRDNT	2	4.558112	-1.55	1490
NSDPFDMVSFKK	2	5.959762	-0.81667	0
HNLKSLTSLEIT	2	6.748744	-0.1	0
SNSNQLIFNWNR	2	9.465071	-1.11667	5500
RVISQPGDAWVT	2	5.836364	-0.1	5500
MNFKSSSSYRYW	2	9.697028	-1.15833	8480
GFLKFPDELASL	2	4.370259	0.425	0
AHPLSWLTSDSM	2	5.078189	-0.08333	5500
GDFYPGSFAVHS	2	5.076711	-0.03333	1490

DNIRSQTALPIN	2	5.836364	-0.58333	0
VNGSNAYTWKAR	2	9.99397	-0.975	6990
NGNLIMEKGSEV	2	4.531398	-0.425	0
FTLNDSIAHVLT	2	5.076711	0.708333	0
VSKLPSGMDVNK	2	8.561547	-0.35833	0
GHNSDMLVYKPA	2	6.742662	-0.54167	1490
TDNVRHTANFYF	2	6.406005	-0.775	1490
HDTKWSLMGQSS	2	6.743628	-1.06667	5500

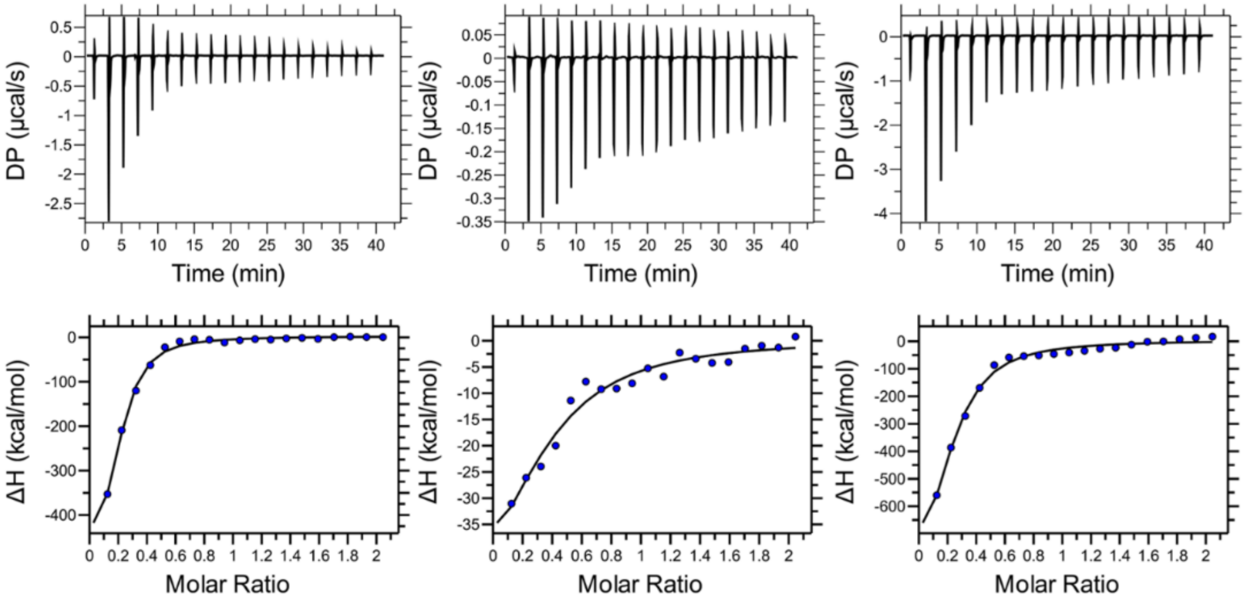

Figure S4: Isothermal Titration Calorimetry (ITC) thermograms for un2p1 binding to MN2 RNA.. Triplicate ITC binding curves evaluating the interaction between un2p1 peptide and unmodified NSUN2 target RNA (UN2) over time (top panels) with integrated heat per injection plotted against the molar ratio (bottom panels).

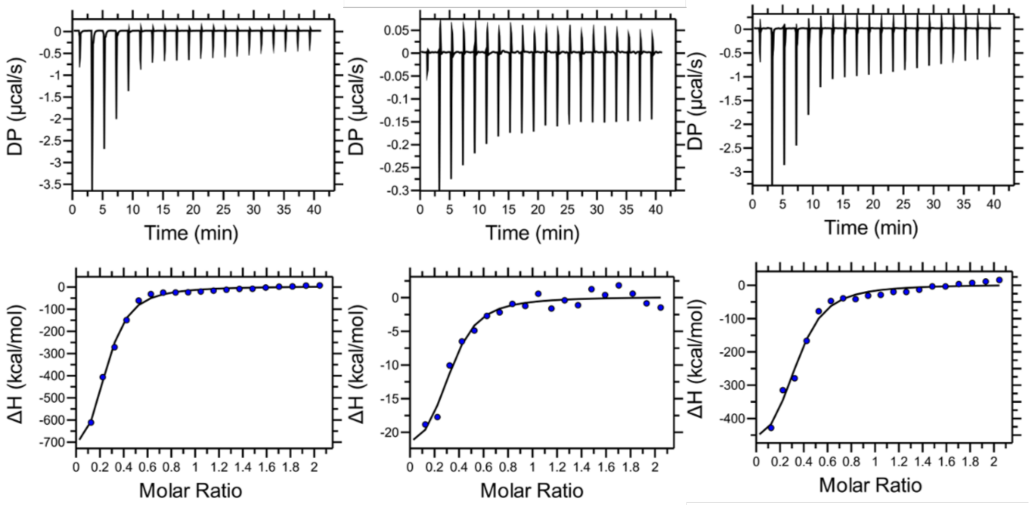

Figure S5: Isothermal Titration Calorimetry (ITC) thermograms for un2p1 binding to UN2 RNA. Triplicate ITC binding curves evaluating the interaction between un2p1 peptide and unmodified NSUN2 target RNA (UN2) over time (top panels) with integrated heat per injection plotted against the molar ratio (bottom panels).

Table S5: Thermodynamic binding constants determined by ITC for un2p1 interactions with target RNAs. Dissociation constants (K_D), stoichiometry (n), and associated fitting error ($\pm\varepsilon$) calculated across triplicate ITC runs for the interaction of peptide un2p1 with m^5C-modified (MN2) and unmodified (UN2) RNA targets.

Sample 1	Sample 2	KD (nM)	± ε	n	± ε
un2p1	MN2 - RNA	1110		4.93	
		556		5.64	
		1670		3.84	
	Average	1010.116	454.79	4.74	0.74

Sample 1	Sample 2	KD (nM)	± ε	n	± ε
un2p1	UN2 - RNA	114		0.218	
		118		0.311	
		108		0.297	
	Average	113.2583	4.11	0.27	0.04

Table S6: BLAST alignment for human proteins homologous to un2p1; BLAST search results identifying sequence alignment metrics (query coverage and E-value) and functional classifications for human proteins sharing homology with selected peptide hits

Description	Query cover	E Value	Accession	Function
HAUS3	58%	1.8	Q68CZ6	Centrosome and Mitotic Protein
ABRAXAS1	75%	1.8	Q6UWZ7	Tumor Suppressor
KNDC1	67%	42	Q76NI1	Signal Transducer / Proto-oncogene Candidate
FSIP2	92%	60	Q5CZC0	Structural / Biomarker Protein
Frizzled-7	50%	60	O75084	Oncoprotein / Receptor: Wnt signaling receptor.
Zinc finger protein	50%	60	Q8NAF0	Transcription Factor.
Alpha-mannosidase 2	92%	86	Q16706	Metabolic / Metastasis-Associated Enzyme
YTHDC2*	75%	86	Q9H6S0	Oncoprotein / RNA Helicase: Reads m^6A RNA modifications to control translation/decay
DRC1	83%	122	Q96MC2	Ciliary / Microtubule Structural Protein
PLCH2	50%	122	O75038	Signal Transducer / Enzymes

(*) m6A reader protein

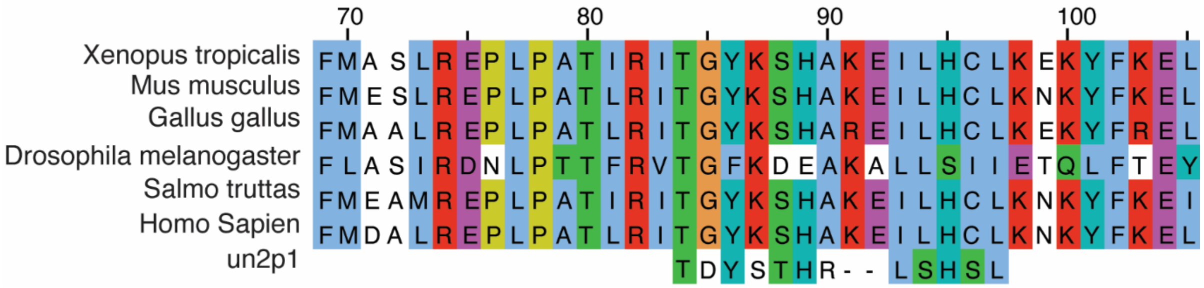

Figure S6: Secondary alignment site for un2p1 peptide in NSUN2 of different eukaryotes is shown; The Primary alignment site shown in Figure 2f.
